# Computational high-throughput screening and *in vitro* approaches identify CB-006-3; A novel PI3K-BRAF^V600E^ dual targeted inhibitor against melanoma

**DOI:** 10.32604/or.2022.025187

**Published:** 2022-10-10

**Authors:** FAISAL HASSAN TOBEIGEI, REEM M. GAHTANI, AHMAD SHAIKH, AMER AL ALI, NADER KAMELI, HOSSAM KAMLI, PRASANNA RAJAGOPALAN

**Affiliations:** 1 Department of Dermatology, College of Medicine, King Khalid University, Abha, Saudi Arabia; 2 Department of Clinical Laboratory Sciences, College of Applied Medical Sciences, King Khalid University, Abha, Saudi Arabia; 3 Department of Clinical Laboratory Sciences, Faculty of Applied Medical Sciences, University of Bisha, Al Nakhil, Bisha, Saudi Arabia; 4 Department of Medical Laboratory Technology, Faculty of Applied Medical Sciences, Jazan University, Jazan, Saudi Arabia; 5 Medical Research Center, Jazan University, Jazan, Saudi Arabia; 6 Central Research Laboratory, College of Applied Medical Sciences, King Khalid University, Abha, Saudi Arabia

**Keywords:** PI3K, BRAF/RAS, BRAF^V600E^, Melanoma, High-throughput screening

## Abstract

Malignant melanoma is characterized by both genetic and molecular alterations that activate phosphoinositide 3-kinase (PI3K), and RAS/BRAF pathways. In this work, through diversity-based high-throughput virtual screening we identified a lead molecule that selectively targets PI3K and BRAF^V600E^ kinases. Computational screening, Molecular dynamics simulation and MMPBSA calculations were performed. PI3K and BRAF^V600E^ kinase inhibition was done. A375 and G-361 cells were used for *in vitro* cellular analysis to determine antiproliferative effects, annexin V binding, nuclear fragmentation and cell cycle analysis. Computational screening of small molecules indicates compound CB-006-3 selectively targets PI3KCG (gamma subunit), PI3KCD (delta subunit) and BRAF^V600E^. Molecular dynamics simulation and MMPBSA bases binding free energy calculations predict a stable binding of CB-006-3 to the active sites of PI3K and BRAF^V600E^. The compound effectively inhibited PI3KCG, PI3KCD and BRAF^V600E^ kinases with respective IC_50_ values of 75.80, 160.10 and 70.84 nM. CB-006-3 controlled the proliferation of A375 and G-361 cells with GI_50_ values of 223.3 and 143.6 nM, respectively. A dose dependent increase in apoptotic cell population and sub G_0_/G_1_ phase of cell cycle were also observed with the compound treatment in addition to observed nuclear fragmentation in these cells. Furthermore, CB-006-3 inhibited BRAF^V600E^, PI3KCD and PI3KCG in both melanoma cells. Collectively, based on the computational modeling and *in vitro* validations, we propose CB-006-3 as a lead candidate for selectively targeting PI3K and mutant BRAF^V600E^ to inhibit melanoma cell proliferation. Further experimental validations, including pharmacokinetic evaluations in mouse models will identify the druggability of the proposed lead candidate for further development as a therapeutic agent for treating melanoma.

## Introduction

Melanoma is an aggressive form of skin cancer that occurs in the melanocytes, caused due to a variety of reasons, often due to the mutations in BRAF, which is responsible for the RAS-RAF-MEK-ERK-MAPK cell signaling pathways [1]. Additionally, it could also be due to the implication of PI3K pathway, which aids and promotes metastasis, leading to an imbalance in cell proliferation and apoptosis [1]. BRAF (v-raf murine sarcoma viral oncogene homolog B1) is a serine/threonine protein kinase that plays a pivotal role in the RAS-RAF-MEK-ERK-MAPK cell signaling pathway. An increased activity of the RAF/MEK/ERK pathway hampers cell cycle progression via a series of phosphorylation events, leading to altered gene expression, cell growth, survival and differentiation in normal and transformed cells [2,3]. Mutations in BRAF also impacts the MAPK pathway, as this pathway play an integral role in relaying of extracellular signals to maintain balance in cellular homeostasis, viz., cell growth and proliferation. Constitutive activation of this pathway as a result of BRAF mutation, viz., V600E, leads to uncontrolled cell growth that leads to tumorigenesis [4]. In several human cancers, including melanomas, PI3K and AKT pathways are found to be abnormally activated and are not regulated resulting in the loss of PTEN tumor suppressor functioning, which is interceded due to several mechanisms that block the mutation and gene amplification, and opposes BRAF inhibition [5]. Studies reveal that PI3CG, the gamma subunit of PI3K and BRAF mutations determine tumor formation and progression due to their active involvement of the RAS genes. Since RAS is primarily involved with the RAF and PI3K pathways, a detailed work on mutated RAS implications is needed to be studied in RAF and PI3K inhibition strategies [6]. Therapeutic management of melanomas have to take into account the efficiency and reliability of single-target therapies. Use of BRAF-inhibitor strategy has poor outcomes as resistance develops via the re-activation of MAPK signaling pathway and/or the activation of PI3K/AKT signaling cascades. The BRAF inhibitors trigger alternate escape routes and alterations occur leading to NRAS and KRAS mutations and over expresses receptor tyrosine kinases [7]. Dual inhibition of both PI3K and BRAF, a combination strategies targeting BRAF and PI3K pathways was found to be effective in suppressing the tumor formation [8]. The use of known and approved BRAF inhibitors such as vemurafenib, dabrafenib are promising initially, since they show progression-free-survival (PFS) and aid in overall survival (OS), but the subsistence is limited due to refractory mechanisms [9,10].

Besides, monotherapy on a long-term treatment regime is restrained and gets hindered due to adversities, such as intrinsic and acquired resistance due to signaling pathway rewiring. These show the rationale for the need of dual targeted therapy while several studies have demonstrated the positive impacts rendered by the combination therapy (CR) as dual inhibitors may act either in coordination or synergistically to counteract the cell toxicity and other adverse effects (AE) in order to create a robust response. The use of adjuvant and supplementary therapy targeting BRAF/PI3K/AKT pathways lowers the risk of relapses and is pivotal for patients with BRAF-mutant melanoma [11–13].

## Materials and Methods

### Materials

All reagents and chemicals were of analytical grade and procured from Sigma Aldrich (St. Louis, MO, USA). Vero, A375 and G-361 cell lines were obtained from the American Type Culture Collection (ATCC, Rockville, MD, USA). Annexin V, cell cycle and PI3K HTRF^TM^ assay kits were procured from Merck Millipore (Burlington, MA, USA). B-Raf^V600E^ enzyme assay kits were from BPS Bioscience (San Diego, CA, USA). FITC-conjugated anti-B-Raf^V600E^ was purchased from Abcam (Cambridge, MA, USA). FITC-conjugated anti-PI3KCD and anti-PI3KCG antibodies were from LS Bio (Seattle, WA, USA).

### Methods

#### Three-dimensional structure retrieval, and processing

The three-dimensional structures of target kinases were retrieved from PDB structure databank, viz., PI3KCG (3ML9), PI3KCD (5T8F), PI3KCA (6GVF), PI3KCB (4BFR), and BRAF^V600E^ (4MNF). Structures were pre-processed by removal of bound crystal waters and addition of polar hydrogens using BIOVIA Discovery Studio Visualizer. Since the available PI3KCG structure has lot of missing hydrogens, for molecular dynamics simulations, full-length PI3KCG structure was retrieved from AlphaFold (AI) based structure database and processed accordingly.

#### Diversity based high-throughput virtual screening (D-HTVS)

For high-throughput virtual screening of large libraries with millions of compounds, e.g., ChemBridge library (1.5 M compounds), ideally ~12 to 18 h for the whole library. Diversity-based high-throughput virtual docking technique developed by SiBioLead LLP, (https://sibiolead.com) was used. For potential kinase inhibitors, ChemBridge compounds (~780,000 compounds) having molecular weight between 350 and 750 kDa were screened. High-throughput docking was performed using Autodock-vina software package with an exhaustiveness of 1 (high-throughput mode). Docking grid box selected based on standard compound bound to PI3KCG (3ML9) or BRAF^V600E^ (4MNF) structures. High throughput docking results and binding poses were confirmed with an addition docking using standard Autodock-vina exhaustiveness.

#### Molecular dynamics simulations

All molecular dynamics simulations were performed using GROMACS simulation package from WebGRO server, available at https://simlab.uams.edu. Briefly, protein-ligand complexes were immersed in a triclinic box containing Simple Point Charge water molecules. Charges in the simulation system was balanced by addition of NaCl as counterions. A further 0.15 M NaCl was added to the system to mimic the physiological conditions. GROMOS96 43a1 forcefield was applied to the system. Simulation system containing protein-ligand complex, water molecules and salt ions were briefly equilibrated before the production run. MD run was conducted for 100 ns using leap-frog integrator. Simulation trajectories were analyzed using GROMACS built-in packages, plotted using XMGRACE software, and visualized in BIOVIA Discovery Studio.

#### Molecular mechanics poisson-boltzmann surface area (MM-PBSA) analysis

Binding free energies for the protein-ligand complex were calculated using GROMACS-based Molecular Mechanics Poisson-Boltzmann Surface Area (MM-PBSA). For MMPBSA calculations last 10 ns frames, 1 frame/ns with a total of 11 frames, including 90 ns frame, were used. Results were analyzed and plotted using MmPbSaStat.py, and MmPbSaDecomp.py utilities.

#### PI3K enzyme inhibition assays

The assay was performed using homogenous time resolved fluorescence (HTRF) based assay kit as per the manufacturer instructions. Briefly, phospotidyl inositol bis phosphate (PIP2) was added to the working reaction buffer that contained desired isoform of PI3K enzyme (delta/gamma) along with appropriate drug and enzyme controls. After adding the desired concentrations of CB-006-3 along with suitable blanks, the reaction mixture was pre incubated for ten minutes at room temperature followed by optimal concentration of ATP. After 30 min incubation at room temperature, the reaction was terminated using stop solution. Detection mixture from the kit was added to all wells, followed by six-hour incubation in dark. HTRF ratio was measured at excitation 337 nM, emission 665 nm, with a delay of 50 micro seconds delay and counting window of 400 micro seconds using FLUOstar Omega microplate reader (BMG Labtech, Ortenberg, Germany). The percentage of inhibition was normalized to control and IC_50_ determined using GraphPad prism software.

#### B-Raf^V600E^ inhibition assay

B-Raf^V600E^ was performed using a luminescence-kinase assay kit as per the manufacturer’s instructions as described elsewhere [14]. In brief, master mix that had 500 µM ATP, Raf substrate, and water were added to 96 well plate with DMSO or test compounds in different concentrations followed by addition of enzyme to initiate reaction. After incubation period of 45 min at 30°C in dark, Kinase-Glo Max reagent was added to wells and luminescence was recorded in FLUOstar Omega microplate reader (BMG Labtech, Ortenberg, Germany). The percentage of inhibition was normalized to control and IC_50_ determined using GraphPad prism software.

#### Cancer cell culture and proliferation assay

Vero, A375 and G-361 were grown as per standard protocols in regular DMEM medium supplemented with 10% foetal bovine serum, 100 U/mL penicillin, and 100 U/mL streptomycin. Cells were passaged two times a week and maintained at 37°C, 5% CO_2_. MTT assay was performed when cells reached 80% confluency using protocol of previously published work [15]. Percentage cell proliferation inhibition was calculated and GI_50_ (half dose for Growth inhibition) was presented with GraphPad Prism 6.0 software. In all cell-based experiments, DMSO was lesser than 0.25% at final concentration.

#### Apoptosis analysis by annexin V assay

Apoptosis in A375 and G-361 cells was computed using an Annexin V detection kit as per the manufacturer’s instructions. Melanoma cells (0.5 × 10^6^) were grown in 6-well plates and treated with desired concentrations of CB-006-3, followed by incubation in 5% CO_2_ at 37°C for 48 h. Post incubation period, cells were harvested, washed with kit buffer, and incubated with 0.25 µg/mL Annexin V reagent for 15 min in the dark. After washing twice, cells were re-suspended in kit buffer containing 0.5 µg/mL propidium iodide. Ten thousand events were acquired on a Guava easyCyte flow cytometer. Data analysis was carried out using InCyte software to differentiate between healthy and apoptotic cells (early and late apoptosis) and presented using GraphPad Prism software (version 6.0; La Jolla, CA, USA).

#### Fluorescence microscopy

Propidium iodide/Hoechst 333258 dual staining was done as recounted elsewhere [16] having a minor change. Both A375 and G-361 cells were grown on cover slip in sterile petri-dish for 48 h. After treatment with CB-006-3, the cells were again incubated for 24 h, cover slips removed, washed with PBS and treated with 2 µl of dye containing 100 mg/ml of propidium iodide and 100 mg/ml of Hoechst 333258 stain. Microscopical analysis was performed using a fluorescence microscope (Nikon, Japan).

#### Cell cycle analysis

The assay was carried out using a cell cycle assay kit according to the manufacturer’s instructions. A375 and G-361 cells at a density of 0.5 × 10^6^ cells/well were seeded in a 6-well plate and incubated for 24 h. After adding 100 nM/200 nM of CB-RAF600E-1, the cells were incubated for a period of 72 h. After washing twice with sterile phosphate-buffered saline (PBS), cells were treated with 50 μL cell cycle assay reagent, incubated in the dark for 15 min, washed twice with wash buffer, and resuspended in HBSS buffer. Ten thousand events were acquired on a Guava easyCyte flow cytometer, and the data were analysed with ExpressPro Software from Millipore (Burlington, MA, USA). The percentage of the cell population at the sub G_0_/G_1_ phase was presented.

#### Inhibition of targeted proteins by flow cytometry

A375 and G-361 cells were treated with 225 or 140 nM of CB-006-3, respectively, and incubated for 4 h in a 5% CO_2_ incubator at 37°C. After incubation, the cells were removed by trypsinization, washed twice with sterile PBS, and re-suspended in HBSS buffer. For B-Raf^V600E^ assay, both cells were treated with 0.50 μg/mL FITC-anti-B-Raf^V600E^ antibody, incubated for 30 min in the dark, washed twice with PBS, and resuspended in HBSS buffer. For PI3K assays, A375 and G-361 cells were treated with 0.7 μg/mL anti-PI3K CD or PI3K CG antibodies were added and incubated for 30 min in the dark. Ten thousand events were acquired using the Guava easyCyte flow cytometer, and the data were analysed with ExpressPro Software from Millipore (Burlington, MA, USA). The percentage of positive cells for each protein was presented.

#### Statistical analysis

Experiments were performed in triplicates and results were expressed as the mean ± S.D. Graph pad Prism 6.0 (from La Jolla, USA) was utilized to carry out statistical analyses. Analysis of IC_50_/GI_50_ values were performed by a non-linear regression fit model with variable slope and accordingly plotted. The differences between groups were analyzed using the two-tailed Student’s *t* test and *p* < 0.05 (*) were considered statistically significant.

## Results

### High-throughput virtual screening of ChemBridge library identify novel PI3K/BRAF^V600E^ binding compounds

**Figure 1:**
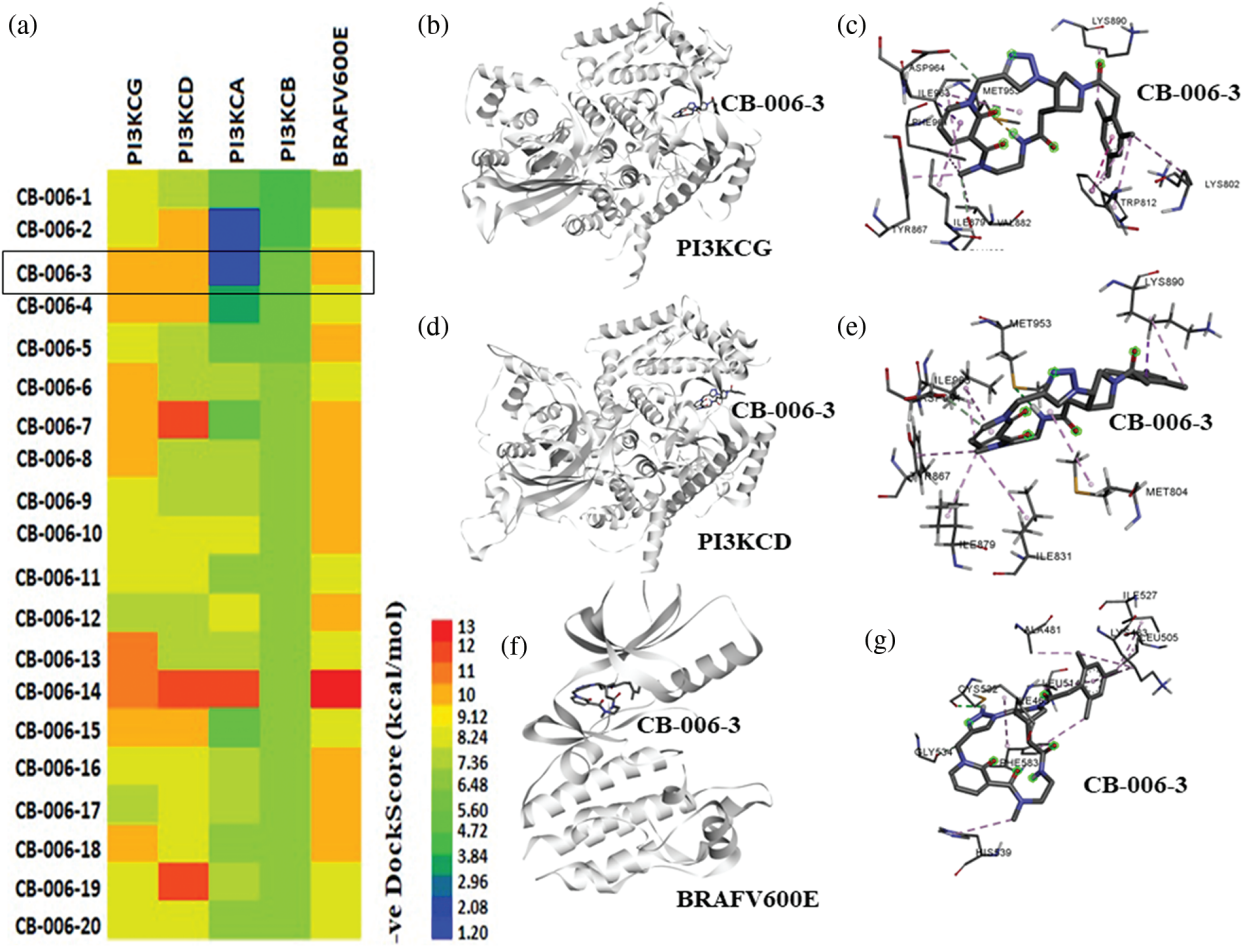
High-throughput virtual screening of ChemBridge database: (a) Heatmap representation of docking energies of ChemBridge compounds with PI3KCG, PI3KCD, PI3KCA, PI3KCB, and BRAF^V600E^ in -ve scale (kcal/mol). (b) CB-006-3 binding with PI3KCG. (c) PI3KCG amino acid residue interactions with CB-006-3. (d) Predicted docking pose of CB-006-3 to PI3KCD and (e) amino acid residue contacts between PI3KCD and CB-006-3. (f) CB-006-3 docking pose with BRAF^V600E^ active site. (g) Protein-ligand interaction analysis depicting BRAF^V600E^ residue interactions with CB-006-3.

In order to identify novel compounds that specifically targets PI3KCG, PI3KCD, and BRAF^V600E^ kinases, we first performed high-throughput virtual screening of ChemBridge compounds, having molecular weight between 350 to 750 kDa, using a diversity-based high-throughput virtual screening technique (see methods for detailed protocol) [14]. We first screened the ChemBridge library against the PI3KCG subunit and identified top compounds with high affinity (stage I screening) for PI3KCG subunit. Identified top compounds were then screened against PI3KCD, PI3KCA, and PI3KCB subunits of PI3K kinase. In order to identify dual inhibitors that specifically target PI3KCG, and BRAF^V600E^ kinases, the top compounds identified from stage I screening were screened against the active site of BRAF^V600E^ kinase. Docking score analysis predicts compounds having high selectivity for PI3KCG, PI3KCD, and BRAF^V600E^, at the same time having low binding affinity for other subunits of PI3K, viz., PI3KCA, and PI3KCB (Fig. 1a). Binding poses and binding score analysis predicts compound CB-006-3, *rel-(17aS,20aS)-19-(mesitylacetyl)-12-methyl-12,13,14,15,17,17a,18,19,20,20a-decahydro-6,10-methano-1,4-methenopyrrolo[3,4-r] [1,2,3,6,12,15]hexaazacyclononadecine-11,16,21-trione*, has a preferential binding for PI3KCG, PI3KCD, and BRAF^V600E^ (Fig. 1a). Compound CB-006-3 as per our requirements has very low binding affinity for PI3KCA, and PI3KCB, indicating a preferential dual inhibitor of PI3K and BRAF^V600E^. Binding pose analysis indicates compound CB-006-3 fits well within the active site and interacts with key amino acid residues involved in the activity of PI3KCG (Figs. 1b and 1c), and PI3KCD (Figs. 1d and 1e), our target PI3K subunits. Similarly, binding pose prediction shows compound CB-006-3 interacts well within the active site of BRAF^V600E^ kinase interacting to key residues (Figs. 1f and 1g). Based on these predictions, we hypothesize that compound CB-006-3 shows preferential binding to PI3KCG, PI3KCD and BRAF^V600E^.

### Molecular dynamics simulation of PI3KCG bound compound CB-006-3

**Figure 2:**
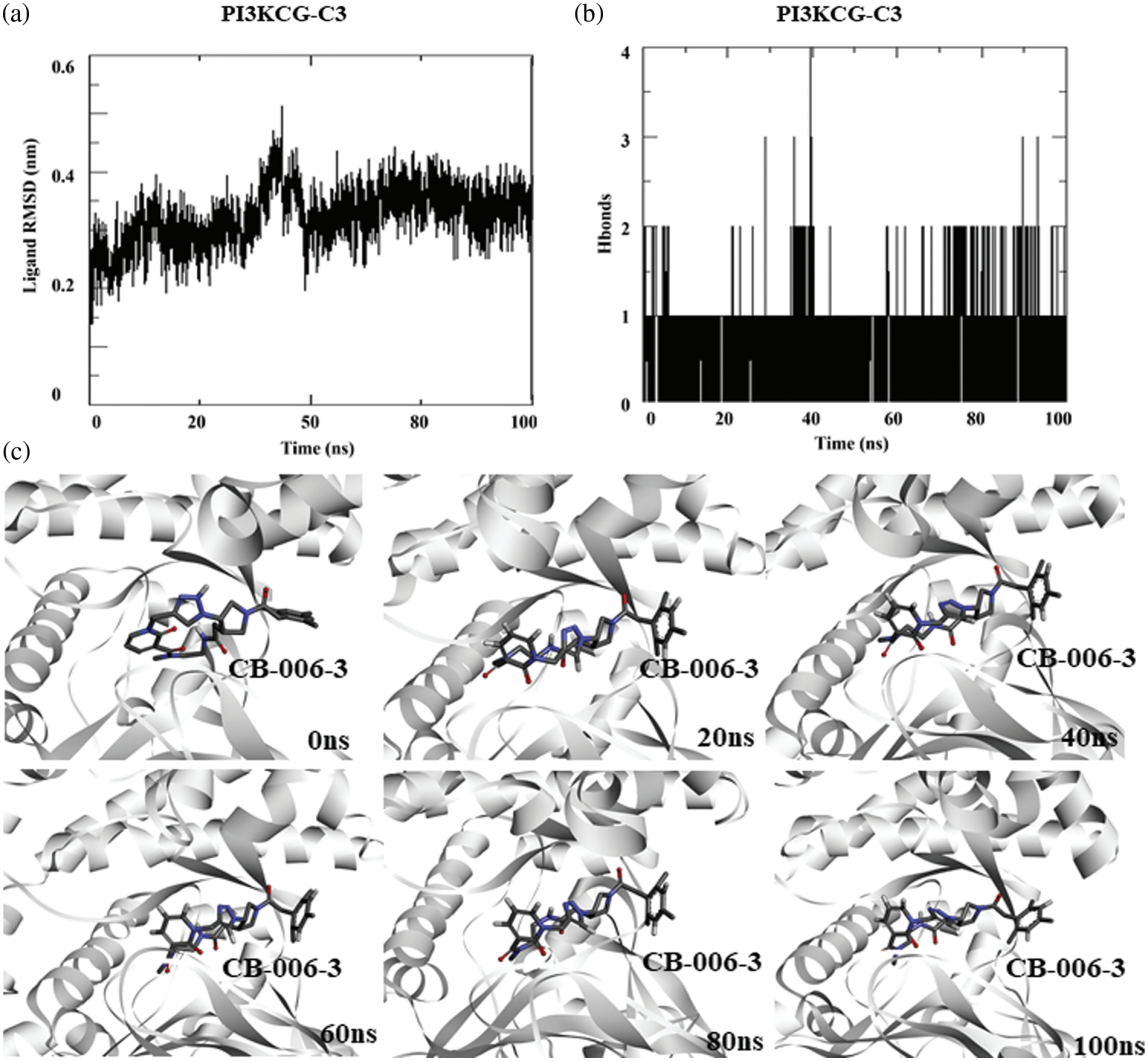
Molecular dynamics simulation of PI3KCG complexed with CB-006-3: (a) Ligand to protein Root Mean Square Deviation (RMSD) for 100 ns trajectories. (b) Number of hydrogen bonds between CB-006-3 and PI3KCG for 100 ns simulation. (c) Snapshots taken at different time points of 0 to100 ns simulation showing binding modes of CB-006-3 with PI3KCG.

As the docking scores predict the relative affinity of a compound binding to a protein, toward understanding the stability of the ligand binding, we performed a 100 ns atomistic molecular dynamics simulation of PI3KCG bound compound CB-006-3 complex, using GROMACS simulation. Since the available crystal structure PI3KCG subunit has missing residues and loops, and this may affect the quality of the simulation, we used the full-length structure of PI3KCG predicted by AlphaFold, an AI based structure prediction method. Docking of compound CB-006-3 with AF-PI3KCG structure showed similar binding compared to our prediction made with experimental structure. We used this complex for MD simulation, and molecular dynamics simulation was performed using our standardized procedure for GROMACS simulations. In brief, the simulation system was filled with Simple Point Charge (SPC) water and ions (NaCl). After a brief equilibration step, simulation was performed for 100 ns. Trajectories were analyzed using in-built GROMACS trajectory analysis scripts. In order to understand the stability of ligand binding, ligand Root Mean Square Deviation (RMSD), and number of protein-ligand hydrogen bonds were calculated and plotted. Results indicate a stable binding of compound CB-006-3 to PI3KCG at the active site. RMSD of ligand to protein implies a stable plot, denoting a stable binding of CB-006-3 to PI3KCG. The number of hydrogen bonds between compound CB-006-3 and PI3KCG remained stable during the simulation (Fig. 2b). We captured protein-ligand interactions at every 20 ns, and the results show a stable binding of CB-006-3 at the predicted binding site, and ligand backbone slightly deviates from initial conformation and remained stable during the simulation, showing that CB-006-3 tend towards a more comfortable binding pose during the simulation (Fig. 2c). Collectively, these results imply a stable binding of predicted compound CB-006-3 to PI3KCG subunit.

**Figure 3:**
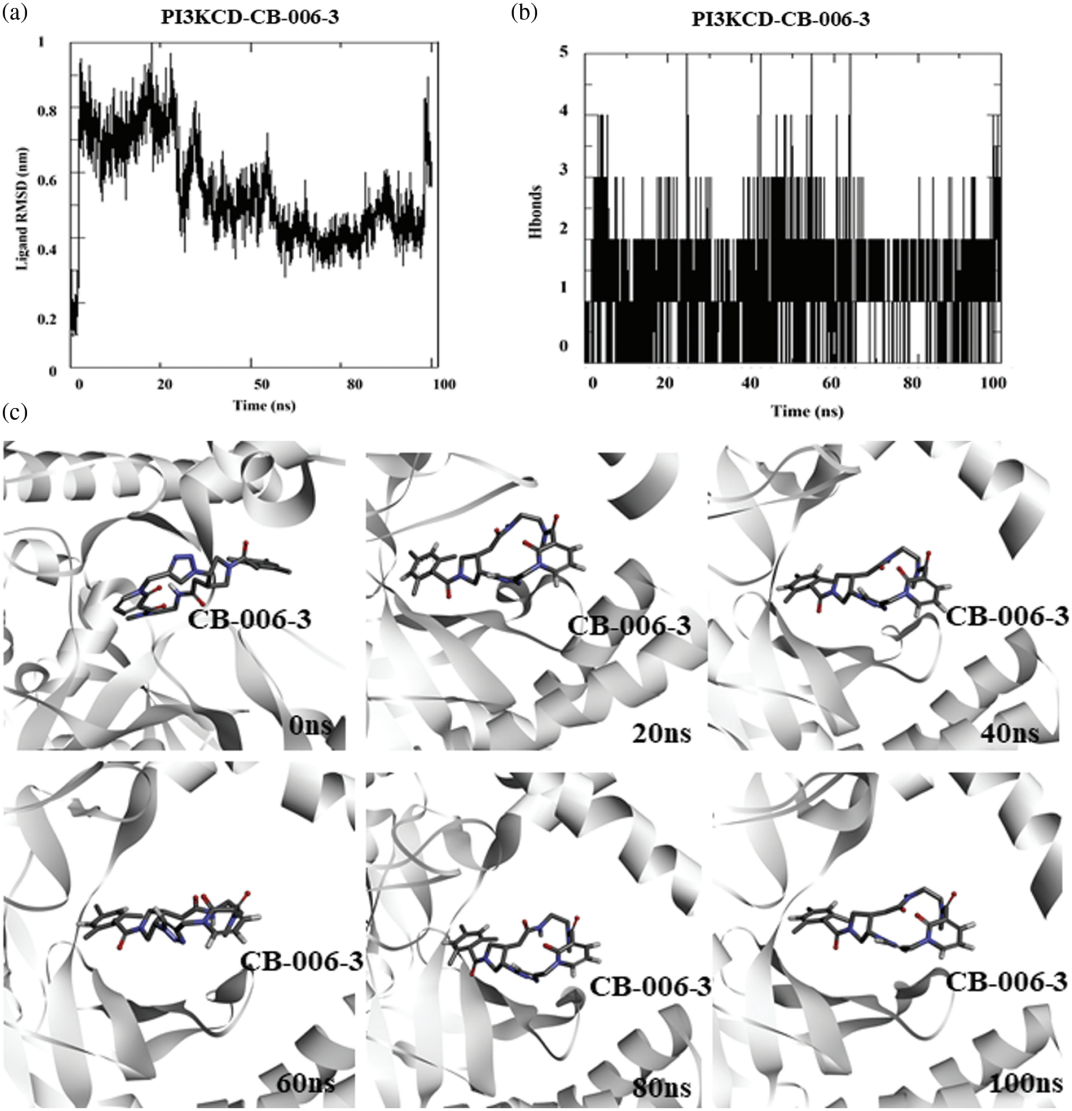
Molecular dynamics simulation of PI3KCD complexed with CB-006-3: (a) Ligand to protein Root Mean Square Deviation (RMSD) for 100 ns trajectories. (b) Number of hydrogen bonds between CB-006-3 and PI3KCD for 100 ns simulation. (c) Snapshots taken at different time points of 0 ns to 100 ns simulation showing binding modes of CB-006-3 with PI3KCD.

Similar to the PI3KCG subunit, we performed MD simulation of PI3KCD subunit bound CB-006-3 complex to determine the protein-drug interaction stability. For simulation, we used the docked complex of Alpha-fold structure of PI3KCD and CB-006-3. Ligand RMSD analysis indicates initial fluctuations in ligand and shows stabilization after 50 ns (Fig. 3a). Average number of hydrogen bonds between CB-006-3 and PI3KCD remained stable during the simulation indicating protein-ligand interaction stability (Fig. 3b). Similar to PI3KCG, in order to visualize the stability of ligand binding, we analyzed simulation frames from different time points. Results indicate CB-006-3 binding remained stable throughout the simulation (Fig. 3c).

### Molecular dynamics simulation of compound CB-006-3 bound to BRAF^V600E^

**Figure 4:**
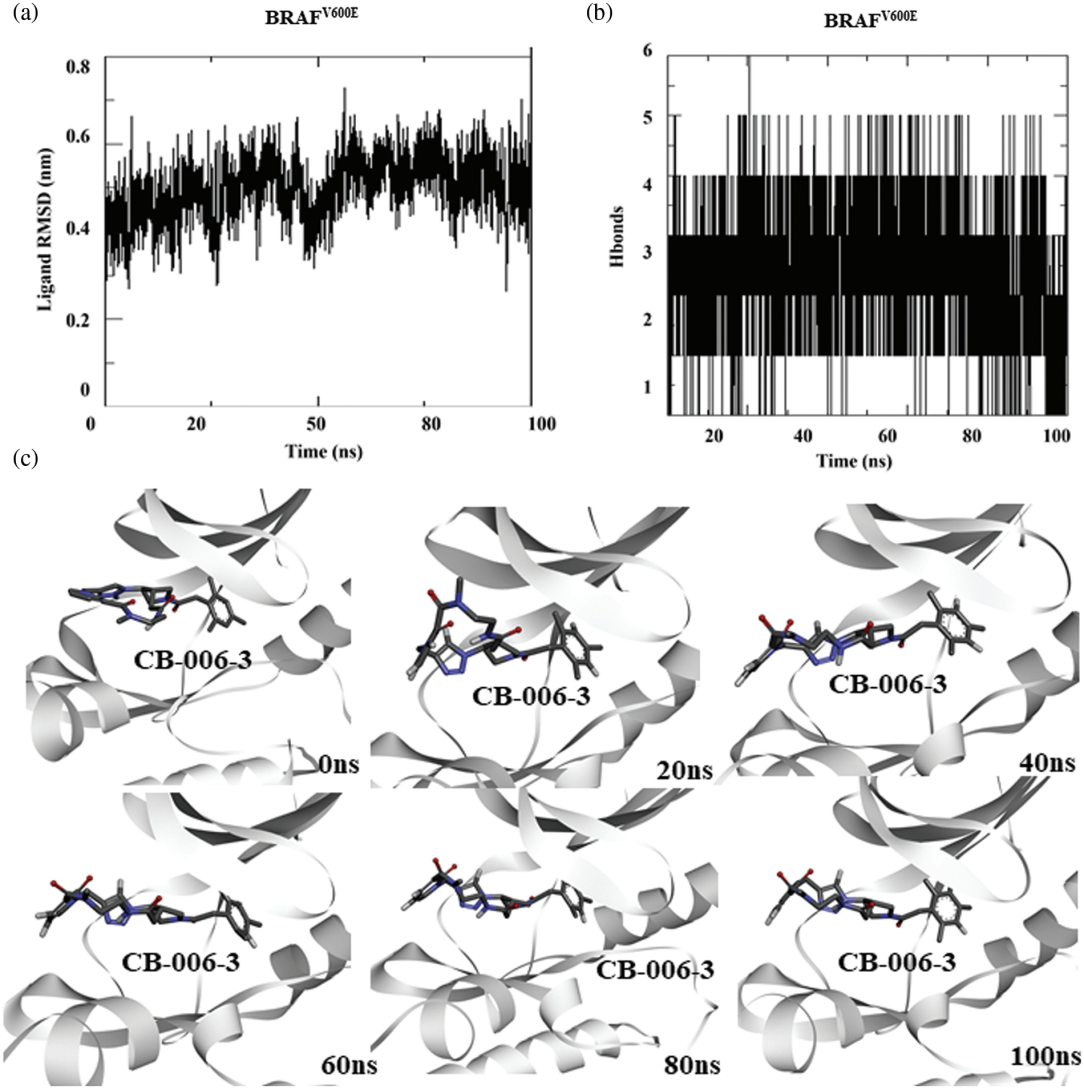
Molecular dynamics simulation of BRAF^V600E^ complexed with CB-006-3: (a) Ligand to protein Root Mean Square Deviation (RMSD) for 100 ns trajectories indicating deviations in CB-006-3 binding. (b) Number of hydrogen bonds between CB-006-3 and BRAF^V600E^ for 100 ns simulation. (c) Snapshots at different simulation time point depicting CB-006-3 interactions to BRAF^V600E^.

Similar to PI3KCG and PI3KCD, toward understanding the stability of compound CB-006-3 binding to BRAF^V600E^ at its active site, we performed 100 ns MD simulation of BRAF^V600E^ bound to compound CB-006-3. As previously explained, simulation box was filled with SPC water and counterions (NaCl). After equilibration, using NVT/NPT method, MD run was conducted for 100 ns. From the simulation trajectories, ligand to protein RMSD and number protein-ligand hydrogen bonds were calculated for each frame in 100 ns simulation. Results indicate a stable ligand to protein RMSD, indicating a stable binding of compound CB-006-3 to BRAF^V600E^ mutant structure (Fig. 4a). Furthermore, the number of hydrogen bonds between BRAF^V600E^ and compound CB-006-3 increases during the simulation, showing the formation of stable and preferred binding between compound CB-006-3 and BRAF^V600E^ (Fig. 4b). Binding pose visualization at different time points in the 100 ns simulation exhibits the compound CB-006-3 binds stably at the kinase (active) domain of BRAF^V600E^ (Fig. 4c). Altogether, these results denote that compound CB-006-3 as a potential dual inhibitor of PI3KCG and BRAF^V600E^ kinase.

### MMPBSA analysis predicts binding free energy

**Figure 5:**
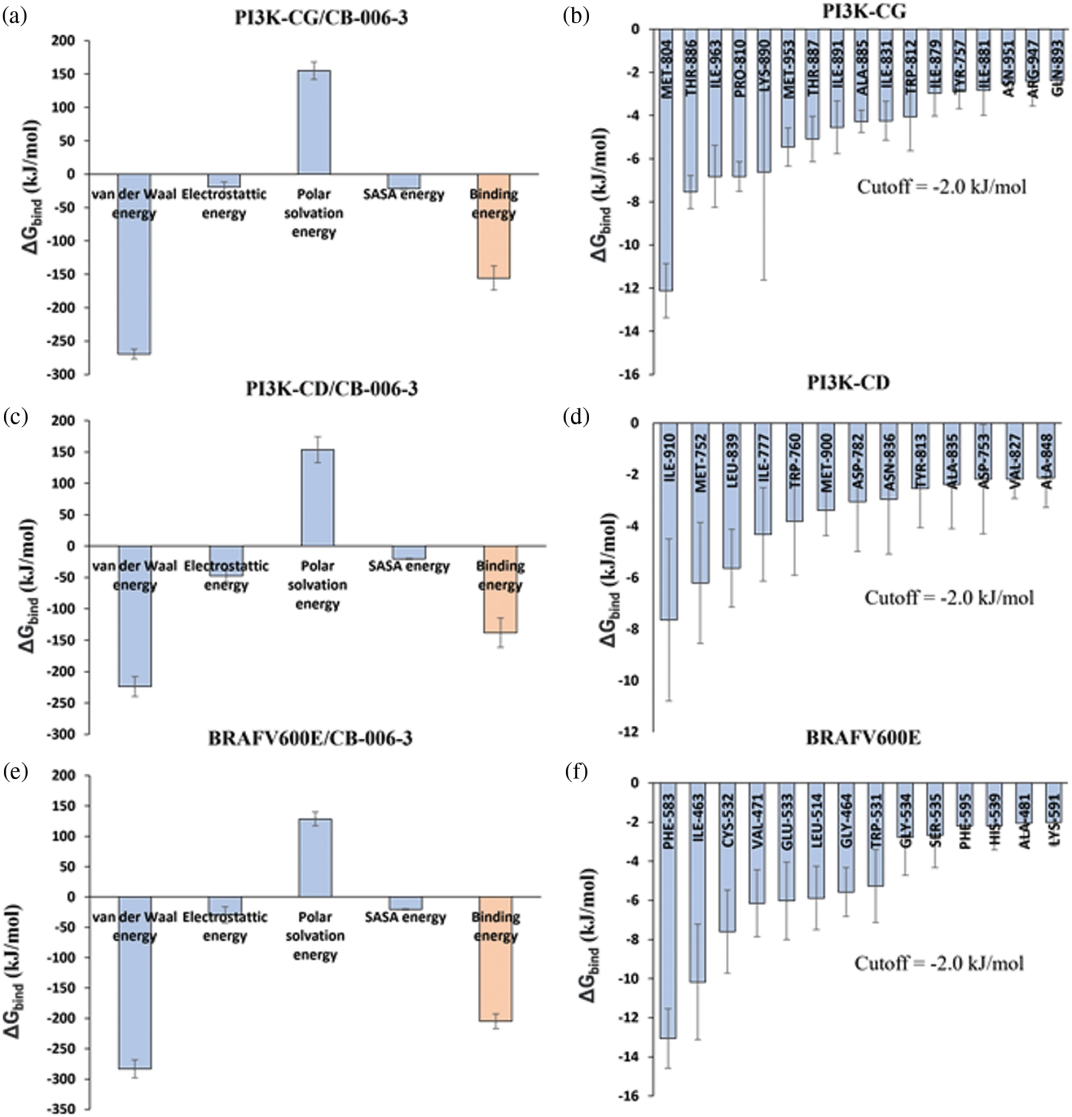
Molecular Mechanics Poisson-Boltzmann Surface Area (MM-PBSA) analysis. (a) Calculated average binding energies (kJ/mol) for the last 10 ns trajectories of 100 ns simulation of PI3KCG complexed with CB-006-3. (b) PI3KCG amino acid residues binding energy contributions to CB-006-3 for the last 10 ns of the 100 ns simulation. (c) Calculated average binding energies (kJ/mol) for the last 10 ns trajectories of 100 ns simulation of PI3KCD complexed with CB-006-3. (d) PI3KCD amino acid residues binding energy contributions to CB-006-3 for the last 10 ns of the 100 ns simulation. (e) Average binding energies calculated for the last 10 ns trajectories of BRAFV^600E^ complexed with CB-006-3. (f) Residue level binding energy contributions calculated for the last 10 ns frames for BRAF^V600E^ and CB-006-3 simulation. Error bars represent standard deviation (n = 3).

We then questioned the likelihood of the binding of compound CB-006-3 to PI3KCG and BRAF^V600E^. Apart from docking score, solvent-based Gibbs binding free energy calculation suggests the prospect of ligand binding to target protein. In order to calculate the Gibbs binding free energy of the protein-ligand complex, we used the last 10 ns trajectories from the 100 ns simulation. Since we conducted our simulations using the GROMACS simulation package, we used the MM-PBSA based approach for calculating Gibbs binding free energies. Results indicate the Gibbs binding free energy of compound CB-006-3 binding to PI3KCG was −155.45 kJ/mol. Fig. 5a shows calculated Van der Waals, Electrostatic, Polar solvation, SASA, and total binding free energies for PI3KCG and compound CB-006-3 complex. From the MM-PBSA calculation, we also evaluated binding energy contributions of PI3KCG residues to compound CB-006-3 binding. Results show Met804, Thr886, Ile963, Ile831, Met953, Pro810, and Lys890, at the active site of PI3K, contribute to a great extent to the predicted binding free energies (Fig. 5b). MMPBSA binding free energy of CB-006-3 to PI3KCD is estimated to be ~−150.0 kJ/mol (Fig. 5c). Residue-based binding free energy contribution indicates residues Ile910, and Met752, and Leu839 contributes to a great extent for the observed CB-006-3 binding energy (Fig. 5d). The BRAF^V600E^-compound CB-006-3 complex also shows a favorable binding free energy. Calculated total binding free energy for compound CB-006-3 to BRAF^V600E^ was −204.97 kJ/mol (Fig. 5e). Other binding energy parameters including van der Waals, Electrostatic, Polar solvation, SASA, shows a favorable energy trend, indicating a preferential binding of compound CB-006-3 to BRAF^V600E^ at the active site. Residue-based binding energy contribution analysis indicates amino acid residues, including Phe583, Ile463, Cys532, Val471, Glu533, and Leu514, contribute to a great extent for the observed binding free energy (Fig. 5f). Based on these analyses, we predict that compound CB-006-3 has a preferential binding affinity and binding stability for PI3KCG and BRAF^V600E^ and can be considered for further validations and development as a dual inhibitor of PI3KCG and BRAF^V600E^, for treating melanoma conditions. Based on these analyses, we predict that compound CB-006-3 has a preferential binding affinity and binding stability for PI3KCG, PI3KCD, and BRAF^V600E^ and can be considered for further validations and development as a dual inhibitor of PI3KCG/PI3KCD and BRAF^V600E^, for treating melanoma conditions.

### CB-006-3 inhibited the PI3K isoforms and BRAF^V600E^ kinases to inhibit melanoma cell proliferations

**Figure 6:**
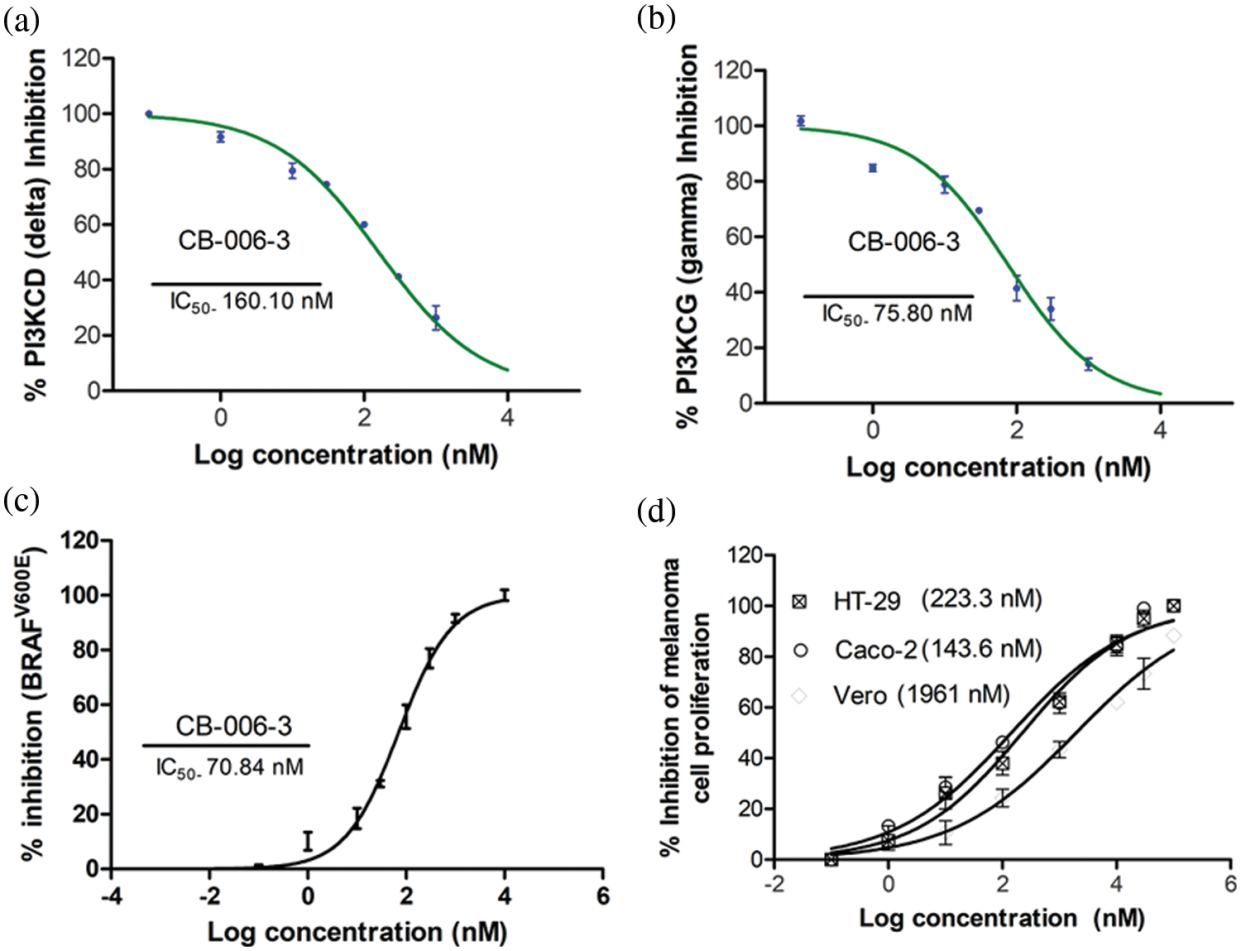
IC_50_ of CB-006-3 against (a) PI3K delta, (b) PI3K gamma and (c) BRAFV^600E^ enzyme forms. (d) (a) GI_50_ of CB-006-3 in controlling proliferation of Vero, A375 and G-361 cells.

In order to verify the *in vitro* efficacy of the observed computational effects, we tested the compound for its inhibition towards the PI3K enzyme isoforms. CB-006-3 effectively inhibited both delta and gamma isoforms of PI3K with IC_50_ values of 160.10 and 75.80 nM, respectively (Figs. 6a and 6b). Further, the compound dose dependently inhabited BRAF^V600E^ enzyme with an IC_50_ value of 70.84 nM (Fig. 6c). In order to evaluate if the enzyme inhibitory effects of CB-006-3 is translated into the anticancer efficacy, we carried out the antiproliferative assay with the compound in A375 and G-361 melanoma cells. As observed in Fig. 6d, the compound inhibited the proliferation of both these cells with respective GI_50_ values of 223.3 and 143.6 nM. The compounds inhibited noncancerous Vero cells at a GI_50_ value of 1961 nM (Fig. 6d), thereby depicting the selectivity towards cancer proliferation inhibition.

### CB-006-3 promoted apoptosis and induced cell cycle changes in melanoma cells

**Figure 7:**
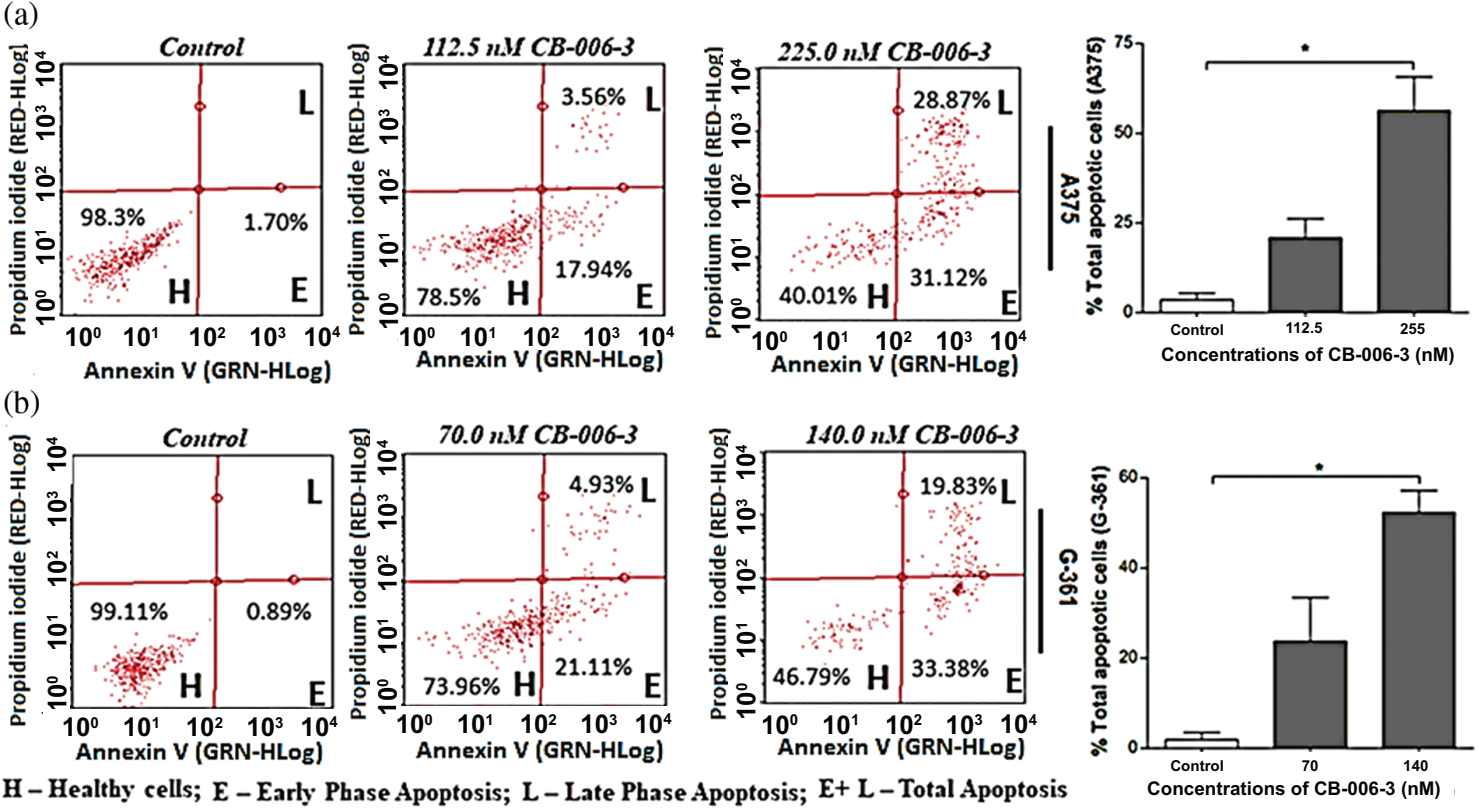
Flow cytometric enumerations in Annexin V staining assay, that show indicating the healthy cells/early and late phase apoptotic cells in (a) A375 and (b) G-361 cells after CB-006-3 treatments. The data were analyzed using InCyte software from Millipore (Burlington, CA USA) and representative figures are presented. Histogram bars represent total apoptotic cell populations as mean ± SD from three experiments and results were statistically significant at *p* ≤ 0.05 (n = 3) compared to * control.

**Figure 8:**
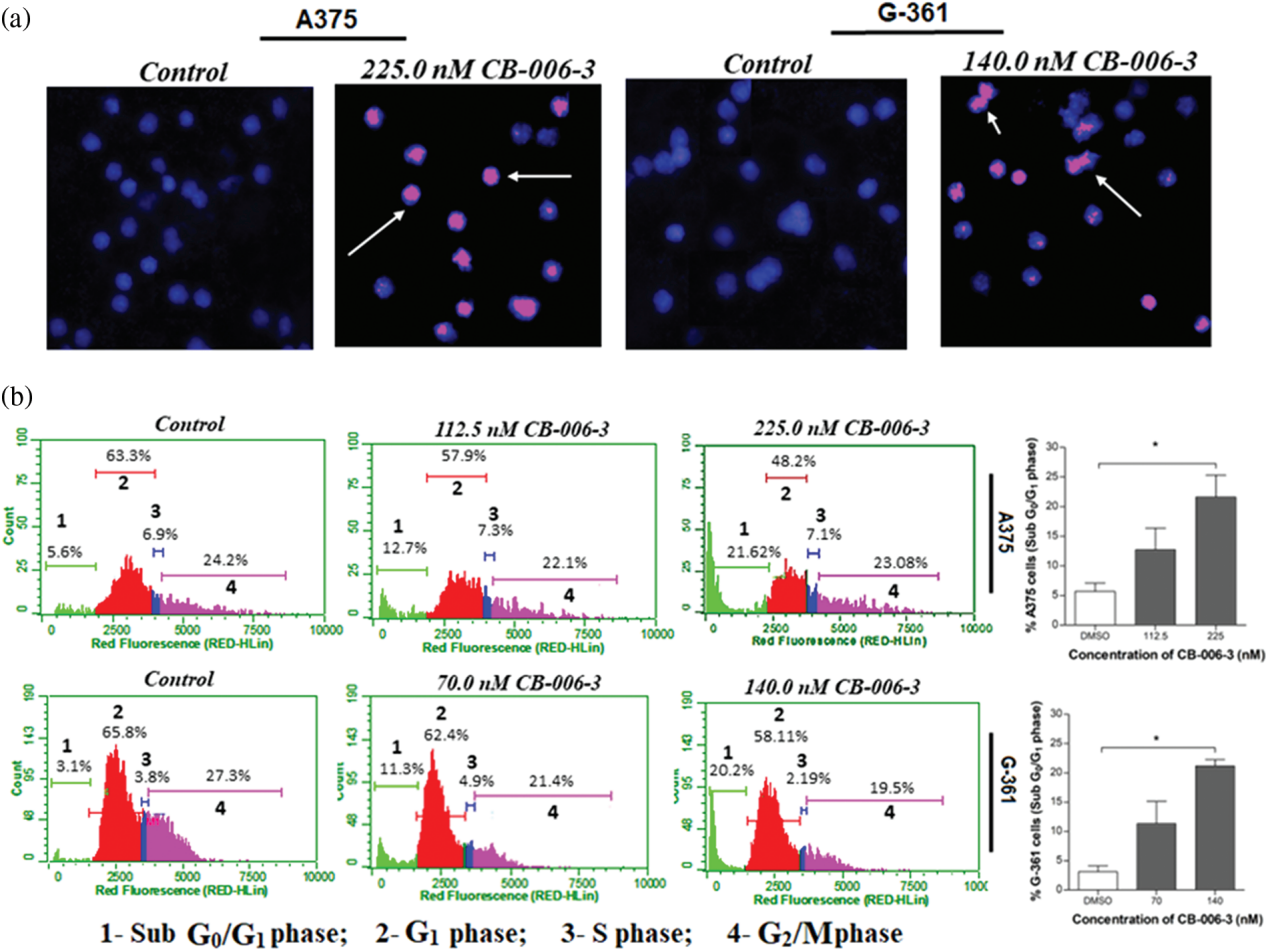
(a) Propidium iodide/Hoechst 333258 dual staining of A375 and G-361 cells after respective near GI_50_ dose CB-006-3 treatments. Arrows pointing the pink color spots indicate condensed/fragmented nuclei bound with fluorescent stain. Microscopic magnification at 200X. (b) Flow cytometry analysis of A375 and G-361 cell cycles with CB-006-3 treatment after 72 h that depicts percentage cell populations different cell cycle phases. The data were analyzed using ExpressPro Software from Millipore, (Burlington, CA, USA) and representative figures are presented. Histogram bars represents mean ± SD percentage of sub G_0_/G_1_ phase cells from three different experiments. Results statistically significant at *p* ≤ 0.05 (n = 3) compared to * control.

To elucidate further if anti proliferative effects could be mediated by apoptotic induction by the compound, we performed Annexin V assay. The nearest GI_50_ values and GI_25_ values of the compound for A375 and G-361 cells were selected as testing concentrations. CB-006-3 effectively increased the percentage of both early and late apoptotic cells in both these cell lines when compared with respective controls (Fig. 7). Further to augment the apoptosis induction on the compound, nuclear staining analysis was carried out in A375 and G-361 cells. Our observations demonstrate clear positivity of nuclear fragmentation in the dual staining analysis, when treated with near GI_50_ concentrations of CB-006-3 in both melanoma cell types (Fig. 8a). Cell cycle analysis revealed appearance of sub G_0_/G_1_ phase cells with increasing concentration of CB-006-3 in A375 and G-361 cells (Fig. 8b). CB-006-3 treatment increased the percentage sub G_0_/G_1_ phase cells to 12.7% and 21.62% with 112.5 and 225 nM concentrations, respectively, while untreated control had 5.68% cells in sub G_0_/G_1_ phase (Fig. 8b). Similarly, 70 nM CB-006-3 treatment to G-361 cells increased the percentage sub G_0_/G_1_ phase cells from 3.1% to 11.30% in comparison to control, which further increased to 20.2% with 140 M compound treatment (Fig. 8b).

### CB-006-3 inhibited the targeted protein expressions in melanoma cells

**Figure 9:**
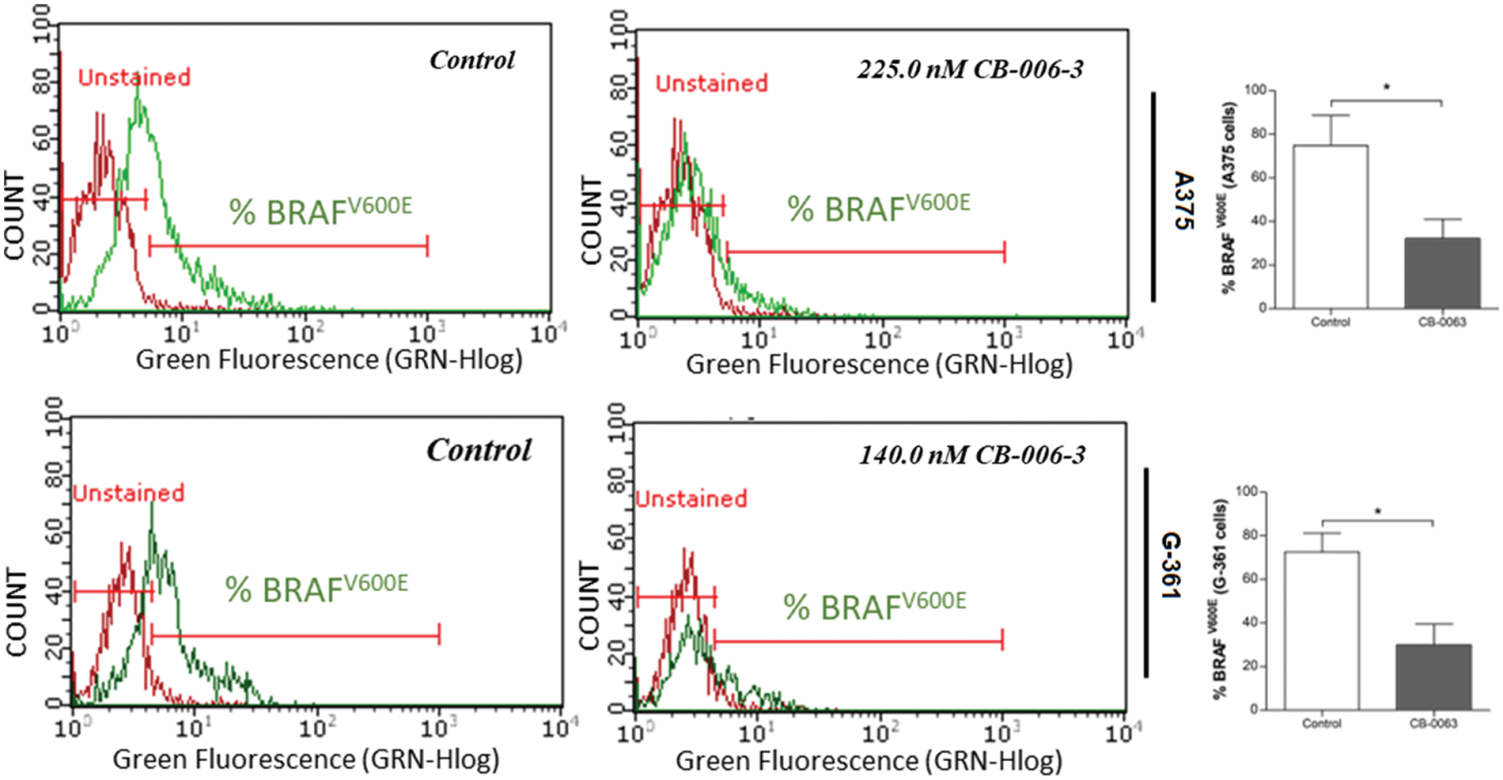
Flow cytometric assessment of B-Raf^V600E^ expression in A375 cells G-361 cells. The data were analyzed using ExpressPro Software from Millipore (Burlington, CA USA) and representative figures are presented. Histograms indicate percentage positive B-Raf^V600E^ cell populations as mean ± SD values from three experiments. Results statistically significant at *p* ≤ 0.05 (n = 3) compared to * control.

**Figure 10:**
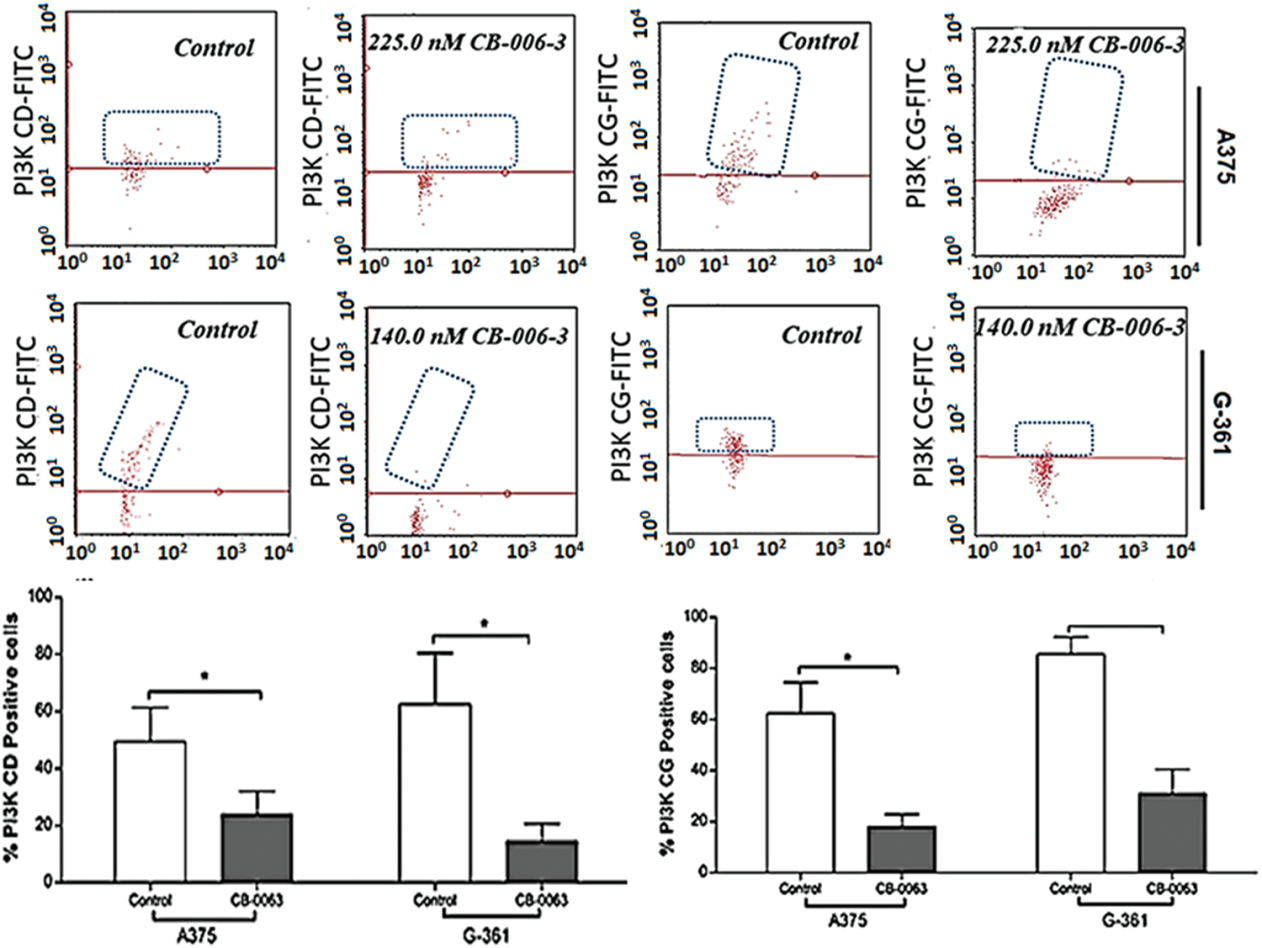
Flow cytometric assessment of PI#K CD and PI3K CG expressions in A375 cells G-361 cells. The data were analyzed using InCyte software from Millipore (Burlington, CA USA) and representative figures are presented. Histograms indicate percentage positive PI3K CD or PI3K CG cell populations as mean ± SD values from three experiments. Results statistically significant at *p* ≤ 0.05 (n = 3) compared to * control.

We investigated the expression status of B-Raf^V600E^, PI3K CD and PI3KCG expressions in A375 and G-361 cells with CB-006-3 treatment. The compound reduced B-Raf^V600E^-positive cell populations from 74.86% in untreated control A375 cells 32.30% in these cells (Fig. 9). A reduction from 72.60% B-Raf^V600E^-positive cells to 30.03% from control to treatment was evident in G-361 cells (Fig. 9). Similarly, CB-006-3 treatment reduced % PI3K CD population from 49.40% to 23.93% in A375 cells and from 62.50% to 14.53% in G-361 cells (Fig. 10). PI3K CG positive pullulations were reduced from 62.35% to 18.16% in A375 cells and 86.41% to 31.09% in G-361 cells (Fig. 10).

## Discussion

Based on computational high-throughput virtual screening followed by *in vitro* analysis, in this work, we predicted compound CB-006-3 as a dual inhibitor of PI3K/BRAF^V600E^ kinases. Previous studies have shown that a majority of melanoma cancers show BRAF^V600E^ activated mutation, therefore targeting BRAF^V600E^ can control cell proliferation to a significant extent [17]. Since the PI3K pathway is also implicated in a majority of the melanomas, studies have shown that targeting both PI3K and BRAF^V600E^ show improved protection as against the traditional chemotherapy agents [18]. We hypothesize that a single small molecule specifically targeting both PI3K and BRAF^V600E^ could reduce multi-drug burden to the patients undergoing melanoma treatment. This work utilized ChemBridge database that comprises more than 1.5 million lead-like compounds. Diversity-based high throughput virtual screening, a new approach for time efficient virtual screening of larger database, identified compounds having high affinity for PI3KCG. According to previous studies, drugs targeting PI3KCG, and PI3KCD has advantages over targeting PI3KCA, or PI3KCB in melanomas [19,20], hence we selected compounds having interactions with PI3KCG and PI3KCD, but not with PI3KCA, and PI3KCB. Since our goal is to ascertain inhibitors having dual targets, i.e., PI3K and BRAF^V600E^, we screened compounds that showed selectivity for PI3KCG and PI3KCD to BRAF^V600E^. Based on the docking results, we found that compound CB-006-3 satisfied our required criteria, i.e., compounds having specific interactions to PI3KCG, PI3KCD and BRAF^V600E^. Protein ligand interaction analysis indicated that CB-006-3 interacts with critical residues at the active (kinase) site of both PI3KCG and BRAF^V600E^. CB-006-3 interacts with residues, including Met804, Thr886, Ile963, Ile831, and Met953 of PI3KCG. Met804 has been proposed as a critical residue involved in ATP binding [21]. Similarly, CB-006-3 showed significant interactions with Phe583, and Val471 of BRAF^V600E^, previous studies emphasized that compounds targeting BRAF^V600E^ active site residues, including Phe583, and Val471, showed selectivity and potency against BRAF^V600E^ mutation. We proceeded further evaluating the binding stability of CB-006-3 with our target kinases. Atomistic molecular dynamics simulation of CB-006-3 complexed with PI3KCG, showed favorable binding stability. Root Mean Square deviation analysis between protein and ligand showed CB-006-3 has a stable binding inside the predicted site at PI3KCG. Furthermore, the number of hydrogen bonds between CB-006-3 and PI3KCG remained stable throughout the simulation indicating ligand stability with PI3KCG. Molecular docking and MD simulation of CB-006-3 binding to PI3KCD, showed, apart from PI3KCG, CB-006-3 also binds to PI3KCD with high affinity. Molecular dynamics simulation of CB-006-3 bound BRAF^V600E^ also showed a stable binding throughout the simulation. RMSD analysis between CB-006-3 and BRAF^V600E^ remained stable, and the number of hydrogen bonds formed between CB-006-3 and BRAF^V600E^ during the simulation increased, indicating that CB-006-3 takes a more comfortable and stable pose at the predicted binding site in BRAF^V600E^. Molecular Mechanics Poisson-Boltzmann Surface Area (MM-PBSA) analysis predicted favorable binding free energy (ΔG binding) between CB-006-3 and PI3KCG. Residue based ΔG binding energy contribution analysis of CB-006-3/PI3KCG complex showed Met804 as one of the primary contributing amino acid residues involved in CB-006-3 binding. Having established that Met804 is a critical residue involved in the activity of PI3KCG, binding of CB-006-3 at the predicted site may block the activity of PI3KCG significantly. Similarly, MM-PBSA analysis of CB-006-3 complexed with BRAF^V600E^ showed a favorable ΔG binding energy. Residue-based binding energy contribution analysis indicated Phe583, and Ile463 as critical contributing residues when targeted by CB-006-3. Thus, based on our rigorous computational modeling and simulation analysis, we predicted CB-006-3 can be pursued as a dual and selective inhibitor of PI3KCG and BRAF^V600E^ kinases for targeting melanomas.

For evaluating our computational predictions that compound CB-006-3 targets PI3K and BRAF^V600E^ selectively, we pursued various *in vitro* assays. The compound had IC_50_ values in nanomolar rage for PI3KCG which was in accordance with the observed computational predictions. CB-006-3 also had activity against PI3KCD, albeit at a higher concentration. Similarly, the compound effectively inhibited the BRAF^V600E^ kinase which was again in line with the computational screening predictions. These *in vitro* enzyme inhibition results were also translated in the melanoma cell proliferation assay, where nano molar range GI_50_ values were observed in both melanoma cell types tested.

When analyzed for the mechanism behind the antiproliferative effect of the compound in melanoma cells, our results indicate increase in the apoptotic cell populations of both A375 and G-361 cells when treated with CB-006-3. These observations were in par with literature, where PI3K [22,23] or BRAF^V600E^ [24] targeted inhibitors individually induced apoptosis in melanoma cells for reducing the cell proliferations. Furthermore, our results also agreed with nuclear condensation and fragmentation, characteristic events of apoptosis for inhibitor treated cells [25,26]. Cell cycle hindrance and appearance of abnormal peaks are considered important check points in cellular death [27]. Appearance of hypo diploid peak in Sub G_0_/G_1_ phase of the cell cycle is a clear indication of the fragmented DNA in cancer cells [28]. These type of abnormalities in cell cycle has also been tightly linked with apoptosis in melanoma cells when treated with inhibitors [29,30]. Treatment of CB-006-3 to melanoma cells and appearance of Sub G_0_/G_1_ phase arrest stands well with these literatures there by conforming the anti-proliferative activity mediated by apoptotic pathway. However, a detailed investigation for determining the pathways involved for the anticancer efficacy by CB-006-3 is recommended as future directives of this study.

## Conclusion

In summary, based on our interdisciplinary approach, we screened the ChemBridge database and predicted CB-006-3 as a dual inhibitor that can selectively target PI3KCG, PI3KCD subunits of PI3K and BRAF^V600E^ mutant kinase to effectively act against melanoma cell proliferations via apoptotic induction. Since both PI3K and BRAF are implicated in melanomas, we believe that targeting both PI3K and BRAF by a single selective inhibitor would be beneficial in reducing drug-induced toxicities. Therefore, CB-006-3 could emerge as a promising lead candidate for treating melanomas with further research for *invivo* and pharmacological profiling.

## Data Availability

Data used in this study is available with the communicating author upon reasonable request for non-commercial purposes.
